# Editorial: Innate immunity: platelets and their interaction with other cellular elements in host defense and disease pathogenesis

**DOI:** 10.3389/fimmu.2023.1292316

**Published:** 2023-09-28

**Authors:** Meganathan Kannan, Firdos Ahmad, Esaki M. Shankar

**Affiliations:** ^1^ Blood and Vascular Biology Research Lab, Department of Biotechnology, Central University of Tamil Nadu, Thiruvarur, India; ^2^ Department of Basic Medical Sciences, College of Medicine, University of Sharjah, Sharjah, United Arab Emirates; ^3^ Infection and Inflammation, Department of Biotechnology, Central University of Tamil Nadu, Thiruvarur, India

**Keywords:** platelet, innate immunity, cell-cell interactions, microparticle, inflammatory responses

Platelets are the anucleate cells that impact the immune system by releasing their cellular contents (1). Immunological reactions are responsible for effectively clearing foreign particles from the body. However, immune responses, at times, can lead to adverse pathological manifestations in the host (2). Platelets play a critical role in innate immunity via encasement of microbial intruders by necessitating the coagulation process, preventing them from disseminating further in the host system. Because platelets can interact with other cellular elements and trigger immune responses either directly or indirectly, they are often regarded as one of the first-line defenders against microbial invasion (3). Further, activated platelets express certain proteins on their surface and release granular contents that provide the ability to cross-talk with immune cells contributing to innate immune responses. When platelets are activated, they release microvesicles called platelet microparticles that play a crucial role in enhancing inflammatory responses (4). These microparticles communicate with immune cells and other cellular elements in the host, thereby contributing significantly to immune responses. Understanding the role of platelet microparticles in the pathogenesis of clinical conditions is essential for effective disease management. This Research Topic has addressed the role of platelets and other associated cellular and soluble elements in host defense and surveillance mechanisms. We invited research related to key areas such as the mechanism and sources of platelet activation, the role of platelets and other cellular elements in defense mechanisms, association of activated platelets in infections, role of platelet activation against viral agents, platelet-microvesicles as immune mediators in health and diseases, Platelet interaction with complement mediators and many other relevant topics. In response to these topics, a total of nine articles were submitted by researchers.

In this Research Topic, Lee et al. proposed that activated platelets promote the expression of CD16+ on CD14+ monocytes. It is known that monocytes and macrophages are the key components of the innate immune system playing a crucial role in the pathogenesis of various inflammatory disorders including rheumatoid arthritis (RA). However, the mechanism of induction of CD16+ on monocytes and its significance remain ambiguous. This study demonstrated that activated platelets play an important role in the expression of CD16 on monocytes, which possibly alters their phenotypical and functional features. Further, this study identified that soluble CD62P, a marker of activated platelets, was elevated in RA patients which justifies the increased expression of CD16+ on CD14+ monocytes in these patients.

Another study by Saris et al. demonstrated that platelets can serve as potential immune modulators contributing to the transfusion-related immune modulation (TRIM) in platelet transfusions. They showed that the platelet releasate inhibits the pro-inflammatory properties of dendritic cells thus decreasing the T cell responses. This study adds value to transfusion-related immune modulation.

Viral infections alter the morphological and biochemical features of platelets to facilitate their participation in defense mechanisms (5, 6). While such alterations of platelets are necessary for the inflammation response, these alterations also trigger hypercoagulation (7). Kim et al. demonstrated that platelet-mediated neutrophil extracellular trap (NET) amplifies coagulopathy that contributes to lung immunopathogenesis during influenza virus A infection. The study strongly indicates that thrombin is a key molecule connecting coagulation and inflammation in infection.

Nanodiamonds (NDs) have potential biomedical applications such as drug delivery and bioimaging (8). Hung et al. tested the induction and rescue of thrombocytopenia in mice with various sizes of NDs and found that those NDs with 50 nm in size promote cell death leading to thrombocytopenia. Further, they found that these NDs induce stronger platelet aggregation, pyroptosis, and apoptosis suggesting the scope for the therapeutic development of thrombocytopenia.

Interleukin 8 (IL-8) is a member of the chemokine family and is one of the early indicators of bacterial infections (9). While IL-8 is secreted by various cells, the mechanism of release was unclear, Quach et al. unraveled the role of platelets in potentiating bacterial-induced IL-8 release from monocytes in human whole blood.

The complete blood count (CBC) parameters, though routine, have become very important in the accurate diagnosis of some of the clinical diseases in the early stage. For example, recent studies have associated dysregulation of mean platelet volume (MPV) with tuberculosis (10, 11). Multiple parameters and cell indices are being associated with clinical outcomes to verify whether these can serve as biomarkers. Weng et al. in this Research Topic, reported that the white blood cell counts to mean platelet volume ratio (WMR) is an important prognostic indicator for acute ischemic stroke outcomes (AIS). The authors studied a total of 549 AIS patients to conclude the above. Additionally, when CBC is combined with the cellular components/releasates, they become critical indicators of various diseases. Urban-Solano et al. studied both latent and active tuberculosis and found that platelet count is correlated very much with the high levels of platelet factor 4 (PF4) and vascular endothelial growth factor (VEGF-A) in active tuberculosis. Baier et al. provided evidence that low platelet count is correlated with the markers of kidney injury and decreased complement C3. The study concluded the link between platelets, complement activation system, and clinical outcome. All three studies reported in this Research Topic strongly suggest that the cellular subpopulations are gaining value in clinical outcomes.

Activated platelets release tiny particles known as platelet microparticles (PMP) that are less than 1 μm in size. These microparticles are thrombogenic and participate in the pathogenesis of various diseases including cancer (12). Elevated PMP, an indicator of activated platelets in circulation, has been identified in several clinical conditions such as hypertension, diabetes, etc (13, 14). Gharib et al. studied the role of PMP in the progression of chronic lymphocytic leukemia (CLL) using cell lines. The study concluded that PMP provokes CLL through metabolic reprogramming.

All the above studies strongly suggest that platelets are the major contributors of the pathogenesis of many clinical conditions. Thus measurement of activated platelets, utilizing a reliable diagnostic tool, in many pathological conditions may undoubtedly benefit the effective management of diseases.

## Author contributions

MK: Conceptualization, Writing – original draft, Writing – review & editing. FA: Writing – review & editing. ES: Writing – review & editing.
